# The effect of metformin and thiazolidinedione use on lung cancer in diabetics

**DOI:** 10.1186/1471-2407-12-410

**Published:** 2012-09-14

**Authors:** Peter J Mazzone, Hardeep Rai, Mary Beukemann, Meng Xu, Anil Jain, Madhu Sasidhar

**Affiliations:** 1 Respiratory Institute, The Cleveland Clinic, 9500 Euclid Ave., A90, Cleveland, OH, 44195, USA; 2 Northside Medical Center, Youngstown, OH, USA; 3 Quantitative Health Sciences, The Cleveland Clinic, Cleveland, USA; 4 Department of Internal Medicine, Cleveland Clinic, Cleveland, OH, USA

**Keywords:** Lung cancer, Diabetes, Metformin, Thiazolidinediones

## Abstract

**Background:**

Metformin and the thiazolidinediones (TZDs) may have a protective effect against the development of lung cancer.

**Methods:**

Patients with diabetes mellitus (DM) were identified from the electronic medical records of the Cleveland Clinic. Diabetics with lung cancer were identified then verified by direct review of their records. Control subjects were matched with cancer subjects 1:1 by date of birth, sex, and smoking history. The frequency and duration of diabetic medication use was compared between the groups. The cancer characteristics were compared between those with lung cancer who had and had not been using metformin and/or a TZD.

**Results:**

93,939 patients were identified as having DM. 522 lung cancers in 507 patients were confirmed. The matched control group was more likely to have used metformin and/or a TZD (61.0% vs. 41.2%, p < 0.001 for any use; 55.5% vs. 24.6%, p < 0.001 for >24 months vs. 0–12 months). In the group with lung cancer, those who had used metformin alone had a different histology distribution than those who received neither metformin nor a TZD, were more likely to present with metastatic disease (40.8% vs. 28.2%, p = 0.013), and had a shorter survival from the time of diagnosis (HR 1.47, p < 0.005).

**Conclusions:**

The use of metformin and/or the TZDs is associated with a lower likelihood of developing lung cancer in diabetic patients. Diabetics who develop lung cancer while receiving metformin may have a more aggressive cancer phenotype.

## Background

Lung cancer is the leading cause of cancer related mortality worldwide. Individuals at risk for developing lung cancer can be identified by clinical epidemiologic factors [1-4]. Recently, molecular predictors of the risk of developing lung cancer have also been sought [5-7]. The only successful means of modifying one’s risk for developing lung cancer is the avoidance of causative exposures, particularly cigarette smoking [8]. Attempts at developing chemopreventive strategies have met with little success to date. When lung cancer is diagnosed, it is often in an advanced stage. Available treatment has a significant impact on outcomes, however, the overall success of treatment remains poor. Advances in the fields of lung cancer chemoprevention and therapy have the potential to reduce lung cancer related mortality.

Patients with diabetes mellitus (DM) often have clinical risk factors for the development of cancer. Suggested links in the biology of DM and cancer include hyperglycemia driving malignant cell growth, the insulin and insulin-like growth factor axis leading to cell survival and mitogenesis, and alterations in inflammatory cytokines leading to suppression of antitumor immunity [9,10]. An epidemiologic link between an increased incidence of various cancers in diabetics, as well as a poorer prognosis for cancer survival amongst diabetics, has been suggested [9,10]. Studies assessing the impact of DM on lung cancer prognosis have yielded variable results [11].

Diabetes treatments may influence the risk of developing cancer and the prognosis of cancer when it develops. There is epidemiologic and pathophysiologic evidence that the biguanide metformin and the class of thiazolidinediones (TZDs) may have cancer suppressing effects [12,13]. The aims of the current study were to determine if the frequency of metformin and/or TZD use differs between diabetic patients who develop lung cancer and those who do not; and to determine if there are differences in the presentation and outcomes of diabetic patients with lung cancer who have used metformin and/or a TZD compared to those who have not.

## Methods

### Study design and data collection

The current study received institutional review board approval from the Cleveland Clinic. This was a retrospective, case–control study. An electronic medical record search of the Cleveland Clinic Health System patients identified subjects with a recorded diagnosis of DM. Data automatically extracted from the electronic record (when recorded) included the diabetic patient’s date of birth, sex, tobacco history, diagnosis of lung cancer, medications being used, date of first use of medications, body mass index (BMI) measures, hemoglobin A1c (HbA1c) values, and whether the subject was living or deceased. Categories of medication use included in the analysis were metformin and/or a TZD vs. neither, metformin without a TZD vs. neither (labeled metformin alone), and a TZD without metformin vs. neither (labeled TZD alone). Insulin and sulfonylurea use was also documented.

For each subject labeled as having a diagnosis of lung cancer in the medical record, the diagnosis was confirmed through a direct review of the documentation within their record. Additional data was extracted from the records of those subjects confirmed as having lung cancer, including the date of lung cancer diagnosis, histology of the cancer, stage at presentation, survival from the date of diagnosis, and details of medication use (type and dates). Lung cancer subjects were considered to have used metformin or a TZD only if the use predated the diagnosis of lung cancer. Survival data was supplemented by use of the Social Security Death Index when necessary.

Control subjects were randomly selected from those who were not labeled as having lung cancer. Control subjects were matched to lung cancer subjects 1:1 based on sex, date of birth (+/− 5 years), and tobacco history when available for the cancer subject (current, former, or never; then pack years +/− 10). When tobacco history was not available for the cancer subject, the control was selected to be a former smoker with a moderate number of pack-years smoked. Eleven lung cancer patients did not have their smoking status documented (current, former, or never). The demographic and cancer characteristics of these 11 were compared to the remainder of the lung cancer patients. There was no difference in any parameter. In addition, data was analyzed both with these 11 (and their matched controls) and without these 11 (and their matched controls) included. There was no difference in any outcome described, thus we chose to include data from the entire group. Additional data collected on the control subjects included details of medication use (type and dates). Control subjects use of metformin or a TZD was analyzed first when defined as use prior to the lung cancer diagnosis of their matched case, and separately when defined as use at any time.

The electronic medical record was available from 2001 through the time of completion of the record review 6/2011. All records available for each patient within this timeframe were reviewed to obtain the above information. The diagnosis of lung cancer could have occurred at any time during the patient’s life, as long as it had been documented definitively within their medical record. The year of diagnosis was available for all cancers (522 of 522, 100%). The date of diagnosis was available for 459 of 522 cancers (87.9%). When the date was not available the midpoint of the year was used. The range of dates of diagnosis was 1978 – 7/28/2010. 24 of the cancers (5.0%) were diagnosed prior to 1995, when the first of the medications in question became available. Data was analyzed both with and without these 24 patients and their matched controls included. Results did not differ between these analyses so we chose to present data from the entire group. The duration of medication use was taken as the date of first documented use to final documented use. If the final documented use was at the time of the last physician visit, the medication was considered to be used up to the point of the end of data collection. Follow-up continued to the point of the last patient visit encounter in the EMR. The SS Death Index was accessed at the end of study data collection.

### Statistical analysis

Continuous variables were assessed by Wilcoxon rank sum tests. Categorical variables were compared using chi-square and Fisher’s exact test where appropriate. Univariate survival analysis was performed using the log rank test with Kaplan-Meier curves. Cox regression was used to assess the hazard ratio for survival in the group with lung cancer after adjusting for statistically significant factors. Conditional logistic regression including all unmatched statistically significant factors, as well as tobacco pack-years was used to assess the influence of metformin and/or TZD use on the risk of developing lung cancer. All analyses were performed with SAS 9.2 (Cary, NC, USA). For comparison between lung cancer cases and matched controls, age was calculated at a fixed point in time (1/1/1990) as matching was based on date of birth. For comparison within the lung cancer group, the age used was the age at the time of lung cancer diagnosis. BMI and HbA1c values used in comparisons were the means of all available values for each study subject, and the median value of the individual subjects mean values for the groups being compared.

## Results

### Patient population

A total of 93,939 subjects with a diagnosis of DM were identified from the electronic medical record search. Of these, 66.4% of all subjects were listed as using metformin and/or a TZD during their life. From the entire group, 645 had a diagnosis of lung cancer listed in their electronic record. Upon review of the electronic charts, a total of 522 lung cancers in 507 subjects were confirmed. The lung cancer and control subjects were well matched for the variables selected, except for tobacco smoking status where unknown values in the lung cancer group were matched to controls with known values (Table 1). The results of the following analysis did not differ if those with missing smoking values (and their matched controls) were excluded from the analysis. The BMI of the lung cancer group was slightly lower than the control group (28.7 kg/m^2^ vs. 30.6 kg/m^2^, p < 0.001). The HbA1c levels of the lung cancer group were similar to those of the control group (7.0 vs. 7.2, p = 0.17).

**Table 1:** Matching of lung cancer and control subjects

		**Lung cancer (507)**	**Control (507)**	**P-value**
**Age**	As of 1/1/1990	56.2 (49.1, 61.6)	56.6 (49.7, 62.0)	0.39
**Gender**	Female	38.9%	38.9%	>0.99
	Male	61.1%	61.1%	
**Tobacco Use**	Current	14.0%	14.2%	0.011
	Former	73.6%	75.3%	
	Never	10.3%	10.5%	
	Unknown	2.2%	0%	
**Pack-years**		40 (25, 45)	35 (25, 45)	0.13
**BMI**		28.7 (25.1, 33.4)	30.6 (26.8, 35.5)	<0.001
**HbA1c**		7.0 (6.3, 7.8)	7.2 (6.5, 7.8)	0.17

Age, pack-years, BMI, and HbA1C are listed as medians (25^th^, 75^th^ percentiles). Age is listed as of 1/1/1990 for comparison purposes since matching was based on date of birth.

All subjects with known diabetes mellitus.

### Association of Metformin and/or TZD Use with lung cancer

Study subjects with lung cancer were less likely to be using metformin and/or a TZD prior to their lung cancer diagnosis than their matched controls (41.2% vs. 61.0%, p < 0.001). Similarly, study subjects with lung cancer were less likely to be using metformin alone (without a TZD), or a TZD alone (without metformin). Study subjects with lung cancer were less likely to be using metformin and/or a TZD for more than 2 years duration than their matched controls (24.6% vs. 55.5%, p < 0.001). (Table 2) In a model including all factors statistically different and/or potentially not completely matched between the cancer and control groups (medication use, BMI, HbA1C, and pack-years of smoking), only medication use was found to be associated with a lower odds of developing lung cancer. The odds ratio (OR) and 95% CI for metformin and/or TZD use is 0.47 (0.32-0.68, p < 0.001), for metformin alone 0.48 (0.28-0.81, p = 0.006), and for TZD alone 0.86 (0.4-1.85, p = 0.14).

**Table 2:** Association between diabetic medication use and a diagnosis of lung cancer

		**Lung cancer**	**Control 1**	**Control 2**	**P-value**
**Metformin and/or TZD**	Any use	41.2	61.0	93.9	<0.001
	>24 months	24.6	55.5	75.1	<0.001
**Metformin alone**	Any use	25.0	30.3	49.5	<0.001
**TZD alone**	Any use	6.5	10.5	9.9	<0.001
**Insulin use**		26.8		33.9	0.014
**Sulfonylurea use**		39.1		42.0	0.34

All numbers expressed as % of subjects taking the drugs listed in each category. Control 1 = started the defined medication prior to the diagnosis of lung cancer in the matched case, Control 2 = started the defined medication either prior to or after the diagnosis of lung cancer in the matched case.

### Lung cancer patient population

In the group of study subjects who had a confirmed diagnosis of lung cancer, those who were receiving metformin and/or a TZD were older than those who were not (72.2 years vs. 68.3 years, p < 0.001). The two groups had a similar sex distribution (% female 37.2 vs. 39.5, p = 0.59). There was a suggestion of more active smokers in the group with lung cancer who were receiving metformin and/or a TZD (18.8% vs. 11.2%, p = 0.099), but a similar number of pack-years smoked was noted between the groups (median 36.5 vs. 40.0, p = 0.68). The BMI was similar in those with lung cancer receiving metformin and/or a TZD and those who were not (BMI of 28.8 vs. 28.3, p = 0.60). The HbA1c was higher in those receiving metformin and/or a TZD (HbA1c of 7.3 vs. 6.7, p < 0.001).

### Lung cancer phenotype associations with metformin and/or TZD Use

**Figure 1:**
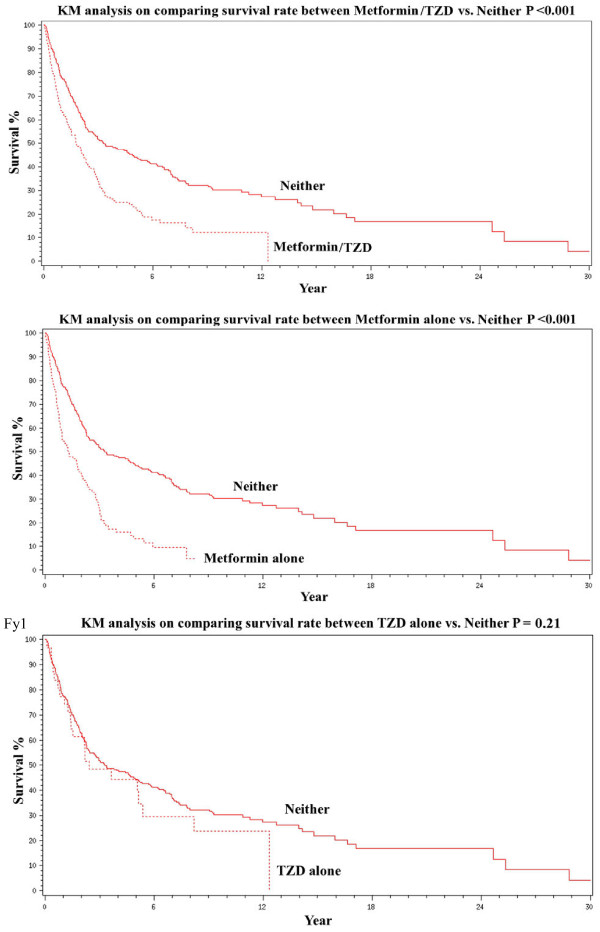
**Survival analysis of patients with DM and lung cancer.A**. Those receiving metformin and/or a TZD versus those who were not. **B**. Those receiving metformin alone versus those receiving neither metformin nor a TZD. **C**. Those receiving a TZD alone versus those receiving neither metformin nor a TZD.

In the group of study subjects who had a confirmed diagnosis of lung cancer, a statistically borderline difference was noted in the lung cancer histology distribution between those who were and were not receiving metformin and/or a TZD (Table 3). This relationship held true for those receiving metformin alone (p = 0.014), but did not reach significance for those receiving a TZD alone (p = 0.82). Those who were receiving metformin and/or a TZD were more likely to present with metastatic disease (39.8% vs. 28.2%, p = 0.008). This relationship held true for metformin alone (40.8% vs. 28.2%, p = 0.013) but not TZDs alone (31.3% vs. 28.2%, p = 0.72). In univariate analysis, the survival of those with lung cancer receiving metformin and/or a TZD was shorter than those who were not (p < 0.001). This relationship held true for those receiving metformin alone (p < 0.001), but not a TZD alone (p = 0.21) (Figure 1). In multivariate analysis, the HRs for survival, after correction for stage and age, in those receiving metformin and/or a TZD was 1.22 (0.96-1.55, p = 0.30), in those receiving metformin alone was 1.47 (1.12-1.92, p = 0.005), and in those receiving a TZD alone was 1.04 (0.65-1.66, p = 0.87) (Table 4).

**Table 3:** Lung cancer histology distribution

	**A. Metformin and/or TZD**	**B. Metformin alone**	**C. TZD alone**	**D. Neither**
**Small cell**	14.2	12.9	14.7	12.2
**Adenocarcinoma**	42.2	47.0	35.3	38.2
**Squamous cell**	23.4	23.5	26.5	19.4
**Large cell**	6.0	3.0	11.8	13.2
**Non-small cell unspecified**	14.2	13.6	11.8	17.1

P-values: A. vs. D. 0.061, B. vs. D. 0.014, C. vs. D. 0.82.

**Table 4:** Hazard ratios for survival in diabetic patients with lung cancer based on their use of metformin and/or a TZD, corrected for stage at presentation and age

	**HR (95% CI)**	**P-value**
Metformin and/or TZD vs. neither	1.22 (0.96, 1.55)	0.30
Metformin alone vs. neither	1.47 (1.12, 1.92)	0.005
TZD alone vs. neither	1.04 (0.65, 1.66)	0.87

## Discussion

Our study has identified an association between the use of metformin and/or a TZD and a lower likelihood of developing lung cancer in patients with DM. In addition, those who developed lung cancer while receiving metformin were more likely to present with metastatic disease, had a lower survival rate (after correction for stage and age), and had a different histology distribution than those who were not receiving either of these drugs.

For an association to be considered causal, several criteria need to be considered. Ideally, there should be a strong association that increases as the exposure increases; the exposure should occur before the disease appears; the association should not conflict significantly with what is already known of the disease; the association should be biologically plausible; and the association cannot be due to any source of error.

The association between the development of lung cancer and the use of metformin and/or a TZD reported here was strong (control group was 1.5 times more likely to have used these medications, OR 0.47) and increased with greater exposure duration (control group was 2.3 times more likely to have used one of these medications for longer than 24 months). All exposures in the cancer group occurred prior to the diagnosis of cancer.

The associations identified in this study are consistent with prior reports. A population based study of 11876 diabetics included 923 subjects with a cancer. Those with cancer were less likely to be receiving metformin than those without (36.4% vs. 39.7%) [14]. In a study of 10309 diabetics, cancer related mortality was reported in 3.5% of metformin users, 5.9% of sufonylurea users, and 5.8% of insulin users [15]. A report of 22,621 females with DM using oral glucose lowering drugs included 305 women with breast cancer. Only 17 of the women with breast cancer had used metformin > 5 years, as compared to 120 matched controls (OR 0.44) [16]. In a neoadjuvant chemotherapy trial for breast cancer 24% of 68 subjects with DM receiving metformin had a complete response to therapy, whereas only 8% of 87 who were not taking metformin, and 16% on non-diabetics did [17]. Metformin use in a mouse model of lung cancer development was shown to reduce the lung tumor burden by 53-72% [18]. Finally, in a cohort trial of 87,678 newly diagnosed diabetics over age 40, 1,371 lung cancers developed over a 7 year timeframe. A 33% reduction in lung cancer risk was seen among TZD users after adjustment for covariates [19].

The associations reported in this study are biologically plausible. Metformin impairs mitochondrial ATP production inducing an energy stress at the cellular level. AMP-activated protein kinase (AMPK) is a key cellular energy sensor. During energy stresses, when the ratio of AMP:ATP increases, AMP binds to AMPK, allowing its activation through phosphorylation by the constituitively active upstream kinase LKB1. Activation of AMPK leads to a shift from energy depleting synthetic metabolic pathways to energy conserving metabolic processes; to an increase in P53 activity, resulting in cell cycle arrest at the G1/S checkpoint, apoptosis, and activation of autophagy pathways; and to a decrease in mTOR activity resulting in a decrease in protein synthesis, cell growth, and a reduction in survivin levels [20-26]. AMPK has a direct link with cell proliferation during the M-phase of the cell cycle, represses mitosis-gene families, and cytokinesis genes [27]. In LKB1 and P53 mutant tumors there may be an inability to compensate for metformin induced energy stresses [25]. Additional potentially protective actions of metformin include decreases in circulating insulin levels, as well as inhibition of phosphorylation of IGF-1R/IR, Akt, ERK, and mTOR [12]. It is possible that lung cancers that develop in the face of metformin use are less sensitive to energy stresses or bypass the pathways influenced by metformin use, resulting in a more aggressive cancer phenotype and the observation of increased metastatic disease, and shorter survival. Thiazolidinediones are ligands for PPAR-gamma, a transcription factor highly expressed in cancer cell lines [28]. Exposure to TZDs in vitro leads to cell cycle arrest, apoptosis, and/or redifferentiation through PPAR-gamma dependent and independent actions [29,30].

The potential for the identified associations to be related to sources of error can never be discounted in retrospective epidemiologic studies. Attempts to minimize the potential influence of random errors and biases were made by including all of the identified lung cancer subjects, having a relatively large sample size, by directly reviewing each of the study subjects’ medical records, and by matching controls based on known and identifiable risk factors. An effect-cause error was avoided by insuring medication use prior to lung cancer diagnosis was documented. The greatest remaining potential for error is the presence of a confounding variable. The level of glucose control and the BMI are potential confounders. The level of glucose control, as estimated by the HbA1c values, did not differ between cancer and control subjects, or within the groups of lung cancer subjects who were and were not receiving metformin and/or a TZD. The BMI of the control group was slightly higher than the cancer group. This is in keeping with other reports suggesting a potential protective effect of obesity on the risk of developing lung cancer [4]. It is difficult to know if the lower BMI in the cancer group represents the protective effect of obesity or if it is related to weight loss from the cancer itself. There was no difference in the BMI of the cancer subjects who were and those who were not receiving metformin and/or a TZD. These factors were not found to be associated with lung cancer risk or survival when assessed with multivariate statistics. There has been a secular trend in lung cancer histology in the United States. An influence of this trend, combined with an increased use of the studied DM medications over time, on the association of histology distribution with medication use cannot be discounted. When only subjects who developed their lung cancer after 1995 were included in the analysis (480 of 507) the results were identical to those presented in the results section (results not shown). Other unknown confounders, such as the potential selection of DM medication based on symptoms or co-morbidities that are associated with lung cancer, cannot be controlled for with this study design. Overall, the strength and consistency of the associations identified support the findings despite the design.

## Conclusions

The use of the biguanide metformin and/or the TZDs is associated with a reduction in the risk of developing lung cancer. Diabetics who develop lung cancer while receiving metformin may have a more aggressive cancer phenotype. The role of these drugs as chemopreventive agents deserves further study.

## Abbreviations

AMP: Adenosine monophosphate; AMPK: Adenosine monophosphate activated protein kinase; ATP: Adenosine triphosphate; BMI: Body mass index; DM: Diabetes mellitus; HbA1C: Hemoglobin A1C; HR: Hazard ratio; mTOR: Mammalian target of rapamycin; OR: Odds ratio; PPAR: Peroxisome proliferator-activated receptor; TZD: Thiazolidinedione.

## Competing interests

The authors declare that they do not have any competing interests.

## Authors’ contributions

PM made significant contributions to the conception and design, acquisition of data, analysis and interpretation of data, drafting and critical revision of the manuscript, statistical analysis, and supervision. He had full access to all of the data in the study and takes responsibility for the integrity of the data and the accuracy of the data analysis. HR made significant contributions to the acquisition of data, critical revision of the manuscript, statistical analysis, and administrative support. MB made significant contributions to the acquisition of data, critical revision of the manuscript, and administrative support. MX made significant contributions to the analysis and interpretation of data, critical revision of the manuscript, and statistical analysis. AJ made significant contributions to the acquisition of data, critical revision of the manuscript, and technical support. MS made significant contributions to the acquisition of data, critical revision of the manuscript, and technical support. All authors read and approved the final manuscript.

## Pre-publication history

The pre-publication history for this paper can be accessed here:

http://www.biomedcentral.com/1471-2407/12/410/prepub
